# Ciliobrevins as tools for studying dynein motor function

**DOI:** 10.3389/fncel.2015.00252

**Published:** 2015-07-06

**Authors:** Douglas H. Roossien, Kyle E. Miller, Gianluca Gallo

**Affiliations:** ^1^Department of Cell and Developmental Biology, University of MichiganAnn Arbor, MI, USA; ^2^Department of Integrative Biology, Michigan State UniversityEast Lansing, MI, USA; ^3^Department of Anatomy and Cell Biology, Shriners Hospitals Pediatric Research Center, Temple University School of MedicinePhiladelphia, PA, USA

**Keywords:** axon, growth cone, tension, microtubule, transport

## Abstract

Dyneins are a small class of molecular motors that bind to microtubules and walk toward their minus ends. They are essential for the transport and distribution of organelles, signaling complexes and cytoskeletal elements. In addition dyneins generate forces on microtubule arrays that power the beating of cilia and flagella, cell division, migration and growth cone motility. Classical approaches to the study of dynein function in axons involve the depletion of dynein, expression of mutant/truncated forms of the motor, or interference with accessory subunits. By necessity, these approaches require prolonged time periods for the expression or manipulation of cellular dynein levels. With the discovery of the ciliobrevins, a class of cell permeable small molecule inhibitors of dynein, it is now possible to acutely disrupt dynein both globally and locally. In this review, we briefly summarize recent work using ciliobrevins to inhibit dynein and discuss the insights ciliobrevins have provided about dynein function in various cell types with a focus on neurons. We temper this with a discussion of the need for studies that will elucidate the mechanism of action of ciliobrevin and as well as the need for experiments to further analyze the specificity of ciliobreviens for dynein. Although much remains to be learned about ciliobrevins, these small molecules are proving themselves to be valuable novel tools to assess the cellular functions of dynein.

## Introduction

Motor proteins are a class of specialized enzymes that transform the chemical energy stored in ATP molecules to mechanical energy. There are three superfamilies of motor proteins (kinesins, myosins, and dyneins), which together total over 100 different motor proteins in humans (Vale, 2003). Though they vary in function, they are all closely associated with cytoskeletal filaments. Dyneins are ATPases which bind microtubules and walk toward the minus end (Sale and Satir, 1977; Paschal and Vallee, 1987; Paschal et al., 1987). Dyneins are divided into three classes. Axonemal dynein is found between microtubule doublets in cilia and flagella and powers microtubule sliding during motility. Cytoplasmic dynein contains two classes, one of which drives transport along microtubules in cilia and flagella (referred to as class 2 or IFT dynein) and another found throughout the remainder of the cell with a variety of functions (class 1) (Vale, 2003). Unless otherwise specified cytoplasmic dynein 1 will herein be referred to as dynein.

**Figure 1:**
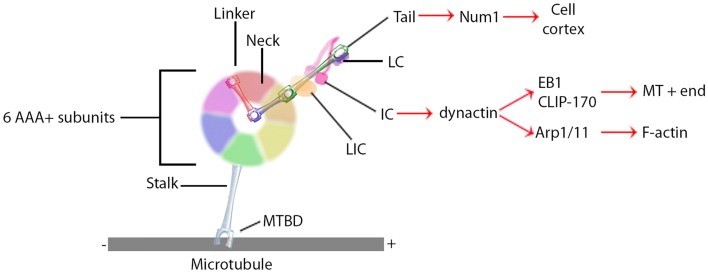
**General dynein complex structure and binding interactions**. Black lines identify components of the core dynein complex whereas red arrows signify binding proteins or localizations. MTBD, microtubule binding domain; LIC, light intermediate chain; IC, intermediate chain; LC, light chain.

**Figure 2:**
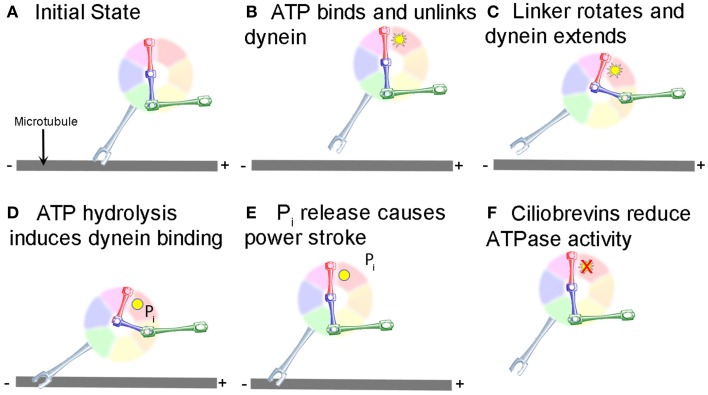
**Mechanochemical cycle of dynein**. **(A)** Dynein is bound to the microtubule in the initial state. **(B)** ATP, represented by the yellow starburst, binds to the AAA+ ATPase domain, causing the MTBD to release from the MT. **(C)** Unlinking of the stalk from the MT causes a conformational change in the linker, which rotates and extends the stalk closer to the minus end of the MT. **(D)** Hydrolysis of ATP to ADP.P_i_ induces binding of the MTBD to the new location on the MT (ADP shown as a yellow circle). **(E)** Release of P_i_ from the motor causes the “powerstroke” and pulls the tail and any attached cargo closer to the minus end of the MT. **(F)** Ciliobrevins, as indicated by the red X, inhibit dynein ATPase activity blocking the step shown in panels **(C,D)**. While this is known to inhibit the motor cycle, how this occurs remains unclear.

Research on the structure and mechanism of dynein function has lagged behind that of the kinesin and myosin motor superfamilies because of its large size and the large number of closely associated regulatory proteins. Recent advances in solving dynein crystal structures have begun to reveal the mechanochemical mechanism beyond what was previously inferred from functional studies. The motor head of dynein is made up of six ATPase domains arranged in a ring (Neuwald et al., 1999). Across this lies a linker domain that changes shape based on the status of ATP binding (Burgess et al., 2003; Kon et al., 2005, 2012; Imamula et al., 2007; Roberts et al., 2009). This linker domain is contiguous with a tail domain, which interacts with a wide variety of regulatory proteins and cargo (Figure 1). A coiled-coil stalk domain emanates from out of the motor head ring and contains a microtubule binding domain (MTBD) at its tip (Gee et al., 1997). When ATP binds to the motor head, the linker domain rotates like a hinge (Roberts et al., 2012) and the MTBD detaches from the microtubule (Porter and Johnson, 1983). ATP hydrolysis then occurs causing the MTBD to bind in a new position further along the microtubule toward the minus end (Carter et al., 2008), which in turn induces ADP + P_i_ release (Holzbaur and Johnson, 1989). This last step in the cycle causes the linker domain to straighten and pull its attached cargo forward as the “power stroke” step (Kon et al., 2005; Kikkawa, 2013) and the mechanochemical cycle can begin again (Figure 2).

How dynein achieves long distance processivity is still unclear, though several lines of evidence suggest dimerization of two heavy chains is responsible for ensuring the motors do not dissociate from the microtubule (Reck-Peterson et al., 2006). The proposed mechanism for dimer function is that stepping of the two dynein heavy chains is uncoordinated and that the presence of the linker between the two increases the probability each will take forward steps as opposed to backward, such that the net result is forward movement of the dimer in 8 nm increments (Reck-Peterson et al., 2006; Dewitt et al., 2012; Qiu et al., 2012). This does not, however, account for the observation that individual heavy chains can processively take 8 nm steps under high load (Mallik et al., 2004), the mechanism of which remains controversial.

Unlike the kinesin and myosin superfamilies, each of which have evolved a large number of subfamilies and isoforms designed to perform specific functions in the cell, the dynein superfamily contains relatively few types (Vale, 2003). Instead, the cell utilizes accessory proteins to adapt the dynein motor complex to numerous cellular functions (Figure 1) (Vallee et al., 2012; Pfister, 2015). These include non-catalytic subunits of the dynein holoenzyme itself; two dynein light chains, a light intermediate chain, and an intermediate chain (DIC). These non-catalytic subunits mostly regulate binding to additional regulatory proteins and cargo (Roberts et al., 2013). Dynactin is a regulatory protein required for long distance dynein-driven transport of materials in living cells (Gill et al., 1991; Schroer and Sheetz, 1991). It is the most commonly studied dynein regulator and is required for almost all known functions of dynein *in vivo* (Schroer, 2004).

Dynactin itself is a large multi-protein complex. The major subunit, p150*^Glued^*, binds to DIC to maintain an intact dynein-dynactin complex (Karki and Holzbaur, 1995; Vaughan and Vallee, 1995). It can also bind microtubules at its N-terminus (Waterman-Storer et al., 1995; Vaughan et al., 2002). This interaction may keep dynein tethered to the microtubule to increase processivity (King and Schroer, 2000). In addition to direct microtubule binding, p150^Glued^ can interact with the microtubule +TIP proteins EB1 and CLIP-170 (Valetti et al., 1999; Vaughan et al., 1999, 2002). Though the precise mechanism is not yet understood, the interactions between dynactin and CLIP-170 target dynein specifically to the plus ends of microtubules, where it can remain in position until cargo binds for transport toward the minus end (Vaughan et al., 2002). Alternatively, the plus end complex can be targeted to the cell cortex along with dynein via Num1 (Markus et al., 2009) or IQGAP1 (Fukata et al., 2002). Lastly, dynactin directly binds the actin related proteins Arp1 and Arp11 (Karki et al., 2000; Eckley and Schroer, 2003), which target the dynein complex to the pointed ends of actin filaments (Eckley et al., 1999; Eckley and Schroer, 2003). Dynactin thus augments dynein function by both increasing the processivity of the motor and regulating its localization.

In addition to localization and cargo binding, dynein regulators can also directly affect motor function. BicD, for example, can increase the velocity of minus-end directed movement of vesicles, though the mechanism is still unknown (Schlager et al., 2014). Originally identified as genes causing brain developmental disorders such as lissencephaly when mutated, Lis1 and NudE have since been shown to have roles in central cell biological processes involving the microtubule cytoskeleton through their interactions with dynein. Lis1 is unique in its ability to bind directly to the motor domain (Figure 1), where it is recruited by NudE to produce a slow yet persistent microtubule-bound force generating dynein state (Yamada et al., 2008; McKenney et al., 2010; Torisawa et al., 2011). Another recent advance in solving three-dimensional crystal structure has shown that Lis1 binds to the AAA+ ring and sterically prevents the linker from completing its conformational change (Toropova et al., 2014). Lis1 has thus been described as a “clutch” (Huang et al., 2012) that keeps the motor coupled to the microtubule yet still able to generate force. In a sense, Lis1 can convert dynein from a mobile cargo transporter to a stable force-producing machine.

## Dynein function in cells

The best characterized function of dynein is for cellular transport. *In vitro*, purified dynein bound to coverslips can move microtubules and dynein in solution can transport plastic beads across stationary microtubules (Lye et al., 1987; Paschal et al., 1987; Euteneuer et al., 1988). In living cells and axons dynein moves membranous vesicles toward microtubule minus ends (Schnapp and Reese, 1989; Schroer et al., 1989). Dynein mediates the transport and cellular distribution of various membranous organelles, such as mitochondria (Pilling et al., 2006), endosomes (Aniento et al., 1993; Driskell et al., 2007), and Golgi (Corthesy-Theulaz et al., 1992). Because most microtubules in the axon are oriented with their minus ends directed toward the cell body, dynein drives axonal transport of membranous organelles in the retrograde direction (Schnapp and Reese, 1989; Pilling et al., 2006; Yi et al., 2011). Dynein also transports short microtubules in the axon via Stop-and-Go transport, though in the anterograde direction (Ahmad et al., 1998, 2006). This is proposed to occur because the dynein motor domain is bound to a short microtubule while the cargo-binding domain is anchored to a structure with more resistance, such as the cross-linked bundle of microtubules or, alternatively, the cortical actin meshwork (Pfister, 1999; Baas et al., 2006). The latter presumably occurs through the interaction between dynactin and Arp1/11. Thus, dynein plays a critical role in the transport of a variety of different types of cargo in the axon and in non-neuronal cells. In all of these cases, the mechanochemical cycle brings the motor head closer to the minus end of the microtubule regardless of whether the microtubule or motor head is stationary.

Other cellular functions have recently been ascribed to dynein in addition to cellular transport, all of which rely on dynein force generation. During mitosis, dynein is required for proper mitotic spindle assembly and alignment (O'connell and Wang, 2000; Rusan et al., 2002; Goshima et al., 2005; Nguyen-Ngoc et al., 2007), capture and alignment of chromosomes (Schmidt et al., 2005), and separation of the centrosomes (Gonczy et al., 1999). Dynein at the actin-rich cortex is presumed to drive a majority of these mitotic functions. By using cortical actin as an anchor, the tendency of the motor to walk toward the minus end moves the microtubule in the plus end direction (Hendricks et al., 2012; Mazel et al., 2013). There is also a growing body of evidence in large cells, such as Zebrafish and *Xenopus* zygotes, that dynein is anchored in the cytoplasm to exert forces on large microtubule arrays (Kimura and Onami, 2005; Wuhr et al., 2010).

Most studies on dynein function in axons have focused on transport, be it Stop-and-Go transport of microtubules or retrograde transport of organelles. There have been, however, a few notable observations made of dynein function in the context of axonal elongation. Overexpression or injection of the dynactin subunit dynamitin disrupts dynein function by dissociating the dynein-dynactin complex (Echeverri et al., 1996; Wittmann and Hyman, 1999). When injected into weakly adhered neurons, the axons lose their ability to resist forces generated by non-muscle myosin II and undergo retraction (Ahmad et al., 2000). Depletion of dynein heavy chain by siRNA makes axons more sensitive to retraction induced by nitric oxide and disrupts growth cone turning (Myers et al., 2006). The rate of axon elongation is drastically reduced with both of these means of dynein disruption as well (Ahmad et al., 2000; Myers et al., 2006). In neurons grown on poly-amine substrates and then treated with soluble laminin, which increases the rate of axon elongation (Lein et al., 1992), there is a dramatic relocalization of dynein to the leading edge of the growth cone that correlates with increases in the rate of elongation (Grabham et al., 2007). These studies indicate that dynein contributes to axonal elongation, in addition to driving retrograde axonal transport. While the importance of dynein to the process of axonal elongation is well-accepted, a difficult issue has been to determine if the primary importance of dynein lies in the sustained delivery of components or if it plays additional roles in axon elongation at the growth cone or along the axon shaft. The primary obstacle to resolving this has been a lack of tools whereby dynein motor function can be acutely and locally disrupted.

## Ciliobrevins inhibit the motor activity of cytoplasmic dynein

Ciliobrevins are a group of small molecules recently determined to be inhibitors of the motor activity of dynein 1 and 2 (Firestone et al., 2012). The discovery of ciliobrevins begun with the identification of a benzoyl dihydroquinazolinone (HPI-4) in a screen of compounds with the ability to impair cellular effects downstream of smoothened signaling in the hedgehog signaling pathway (Hyman et al., 2009). Interestingly, prolonged treatment with HPI-4 was also observed to decrease the number and length of cilia. To follow up on this observation, Firestone et al. (2012) synthesized analogs of HPI-4 and found a group that inhibited hedgehog signaling but did not affect cilia, and one group which affected both. The latter group was termed the ciliobrevins (A-D). The hedgehog pathway drives the accumulation of a signaling component termed Gli2 at the tip of cilia. Ciliobrevin A and D were found to mimic the effects of the N-terminal domain of hedgehog on the targeting of Gli2 to the tips of cilia. This observation suggested to Firestone et al. (2012) that ciliobrevins may be affecting aspects of the intra-flagellar/cilial transport of Gli2. Kinesins and dynein are respectively considered to mediate the anterograde and retrograde transport of flagellar/cilia components. Loss of dynein 2 had been previously reported to result in increased levels of Gli2 in cilia, as observed with ciliobrevins, suggesting ciliobrevins may be interfering with dynein function. Using *in vitro* reconstituted systems for tracking the motor activity of dynein, as reported by the motor protein's ability to move assembled microtubules, ciliobrevin A and D were found to block dynein activity. In contrast, ciliobrevins had no effect on kinesin-1 mediated microtubule movements. Analysis of the ATPase activity of dynein and kinesin 1 and 5 revealed that ciliobrevin D affects dynein ATPase function, but not the tested kinesins. Hanes-Woolf analysis indicates that ciliobrevins may act as ATP competitors for the dynein ATPase (Figure 2F). Ciliobrevins did not affect the binding of dynein to microtubules in the ADP-loaded state. Ciliobrevins were also shown to be effective in living cells, and blocked a variety of phenomena considered to be dependent on dynein 1 or 2. Ciliobrevins inhibited melanosome aggregation in Xenopus melanophores, peroxisome movement in insect cells, and disrupted spindle pole assembly and kinetochore microtubule attachment in NIH-3T3 and HeLa cells. Collectively, the work by Firestone et al. (2012) provides compelling evidence that ciliobrevins A and D are effective inhibitors of dynein activity and can be used to assay dynein functions in living cells.

## Utilization of ciliobrevins to probe the functions of dynein in non-neuronal cells

Since their initial discovery and characterization, ciliobrevins have been used by multiple groups to probe the functions of dynein in living non-neuronal cells. Kim et al. (2014) used ciliobrevin A to determine the role of dynein in the targeting of smoothened into cilia. Consistent with the work of Firestone et al. (2012), ciliobrevin initially decreased the efflux of smoothened from cilia, while longer term treatments also impaired to targeting of smoothened into cilia. Cao et al. (2015) used ciliobrevin D to investigate the role of dynein in the targeting of the membrane protein SAG1-C65 to the periciliar domain of *Chlamydomona*. Sikirzhytski et al. (2014) used ciliobrevin D at sub-maximal doses to decrease, but not completely inhibit, dynein activity during mitosis. This resulted in normal bipolar spindles and chromosome congression into the metaphase plate, but increased the width of the spindle pole. Fu et al. (2014b) used ciliobrevin D to inhibit cilia formation and concluded that cilia are necessary for the differentiation of muscle cells. Eyre et al. (2014) used ciliobrevin D to investigate the role of dynein in the control of the subcellular distribution of the hepatitis C viral protein NS5A, which is required for efficient viral RNA replication and infectious viron assembly. This study revealed that dynein is required for the intracellular traffic of NS5A and efficient viral RNA replication. Yi et al. (2013) used ciliobrevin D to determine that the reorientation of the centrosome toward the immunological synapse in T cells depends on a microtubule dependent and dynein driven mechanism. In contrast, Liu et al. (2013) found that while inhibition of dynein or myosin II alone, using both pharmacological (ciliobrevin D and blebbistatin, respectively) and molecular approaches, generated only mild effects on centrosome positioning toward the immunological synapse, inhibition of both motors caused much more pronounced effects.

Recent evidence indicates that dynein also has roles in the orchestration of active signaling mechanisms in cells. Clippinger and Alwine (2012) used ciliobrevin to determine that dynein transports mTOR to the perinuclear domain. The lack of mTOR transport results in impairment of mTOR activity due to the failure to localize it in the proximity of the upstream activator RheB. Using lymphocytes, Wang et al. (2015) investigated the role of dynein in the internalization and centripetal transport of CD40 following binding to its ligand CD154. Ciliobrevin D treatment impaired the internalization and transport of CD40, as well as the activation of the downstream MAPK pathway by CD154-CD40.

Investigations with ciliobrevins are contributing to the growing literature indicating an interdependence between anterograde and retrograde transport mechanisms. Blasius et al. (2013) addressed the role of retrograde transport mechanisms in the regulation of the accumulation of kinesin-1 motors in the distal processes of differentiated CAD cells. Acute treatment with ciliobrevin A or D decreased the levels of kinesin-1 at the distal tips of processes. This observation is counter to the expectation of increased levels of distal kinesin-1 if retrograde transport mediated the evacuation of kinesin-1 from the tips of processes, indicating that retrograde transport does not have a major role in maintaining the distal accumulation of kinesin-1. However, the observation indicates a possible impairment of the anterograde transport and delivery of kinesin-1, which was further suggested by additional considerations in Blasius et al. (2013). Similarly, Ye et al. (2013) found that ciliobrevin treatment rapidly (2–3 min) inhibited the intraflagellar retrograde transport of the intraflagellar transport component IFT88, but at longer times following treatment (30 min) also inhibited its anterograde transport. In a study of the role of intraflagellar transport mechanisms in cilia-mediated *Chlamydomonas* gliding motility, Shih et al. (2013) also observed that ciliobrevin D treatment affected both retrograde and anterograde intra-flagellar transport. The bidirectional effects of inhibiting dynein and treatment with ciliobrevin D on microtubule based transport mechanisms are further considered in the next section. Collectively, these studies emphasize the usefulness of ciliobrevins as tools for addressing the cellular functions of dynein in a variety of cellular model systems.

## Use of ciliobrevins to address the role of dynein in primary neurons

The dynein-dependent retrograde transport of organelles and proteins from the distal axon to the cell body is a fundamental aspect of neurobiology, and the malfunction of transport mechanisms is considered to underlie aspects of neurodegenerative conditions (Ikenaka et al., 2012; Kanaan et al., 2013). Two studies on the intra-axonal translation of proteins with functions in the cell body have used ciliobrevins to block their subsequent retrograde transport. Melemedjian et al. (2014) provided evidence that IL-6 treatment of the distal axons of sensory peripheral nerves *in vivo* induces the intra-axonal synthesis of the transcription factor CREB, which subsequently undergoes retrograde axonal transport along the nerves and contributes to the development of pain sensitivity. In this study, ciliobrevin D was locally delivered along peripheral nerves, proximal to the site of IL-6 treatment, and found to block the IL-6 induced pain sensitivity. Similarly, local delivery of microtubule depolymerizing drugs, which also perturb transport, blocked the development of pain sensitivity. Control experiments indicated that the effects of ciliobrevin D and microtubule depolymerizing drugs were indeed local. Similarly, Baleriola et al. (2014) report that Amyloid β-peptide 1-42, considered to contribute to the development of Alzheimer's disease, induced the intra-axonal synthesis of the transcription factor ATF4 which in turn is a component of the ensuing cell death mechanism within the neuronal cell body. Treatment with ciliobrevin A specifically to axons using microfluidic chambers prevented the retrograde transport of axonally synthesized ATF4 and decreased Amyloid β-peptide 1-42 induced cell death. These studies provide evidence that ciliobrevins provide a new venue for investigating the role of retrograde axonal transport in experimental paradigms involving acute and localized treatments.

Two studies using cultured embryonic sensory neurons report that treatment with ciliobrevin D causes the rapid, and reversible, cessation of axon extension (Roossien et al., 2014; Sainath and Gallo, 2014). Local application of ciliobrevin D to the growth cone, using microperfusion, was sufficient to stall the extension of the axon, indicating a role for dynein at the growth cone during axon extension (Roossien et al., 2014). The main axon also generates collateral branches which assist in the formation of complex circuitry. Ciliobrevin D inhibited the formation of axon branches in response to the branch inducing signal nerve growth factor (NGF; Sainath and Gallo, 2014). Roossien et al. (2014) also noted that, similar to inhibition of dynein activity through the intracellular injection of function blocking antibodies, ciliobrevin D treatment inhibited the en bloc translocation of the axonal microtubule array, as deduced from the motion of docked mitochondria. In the same study, treatment with ciliobrevin D also increased axon tension, providing insights into the effects of dynein inhibition on axon extension. The effects of the local and acute inhibition of dynein function, achieved through treatment with ciliobrevin in these studies, further agues for a role of dynein in regulating axon extension through mechanisms operative within the axon itself.

Interestingly, ciliobrevin D treatment impaired both the retrograde and anterograde transport of mitochondria (Roossien et al., 2014; Sainath and Gallo, 2014) and lysosomes and Golgi-derived vesicles along axons (Sainath and Gallo, 2014). The bidirectional effects of altering dynein function on transport are not novel, and interested readers are directed to the discussion in both publications. The novelty of the observations using ciliobrevin D in the context of the role of dynein in bidirectional transport is that they indicate that inhibition of the ATPase activity of dynein alone may translate into bidirectional effects on transport. In contrast, a study of the *initial* emergence of neurites from the cell bodies of cultured *Drosophila* neurons failed to note effects of ciliobrevin D treatment on neurite formation (Lu et al., 2013). The initial formation of neurites from the cell bodies of these neurons was found to depend on kinesin-1 driven microtubule gliding into the nascent processes. In this study ciliobrevin D impaired the kinesin-1 dependent motility of mitochondria as noted in the studies by Roossien et al. (2014) and Sainath and Gallo (2014), but did not affect kinesin-1 mediated microtubule sliding. The observation that ciliobrevin D affects one form of kinesin-1 driven motility, but not another, is interesting and indicates that the bidirectional effects of inhibiting dynein may not translate to all cargoes. One possibility that may explain these differences is that in some cases kinesin and dynein activity are regulated cooperatively; such that when one motor is active the other is inhibited (Miller et al., 2005; Fu and Holzbaur, 2014a; Hancock, 2014). Taking this idea to its logical conclusion raises the possibility that when ciliobrevin binds to dynein it causes it to assume a physical confirmation that mimics an “active” motor and thus suppresses kinesin. Yet in other cases, there is a “tug-of-war” between kinesin and dynein where the activity of the motors is not coordinated and thus disruption of dynein allows kinesin to win. Another possibility is that when kinesin and dynein work cooperatively, there is a checkpoint mechanism in place that ensures that functional kinesin and dynein are both bound to the cargo. Thus, if either kinesin or dynein are absent or dysfunctional, the checkpoint is flagged and the cargo is motionless. If this occurred ciliobrevin may inhibit bi-directional transport by either causing the disassociation of dynein from the motor complex or by causing dynein to assume a confirmation that is “non-functional.” Finally, it is possible that ciliobrevin locks dynein in a confirmation where it is stably associated with microtubules but is unable to generate forces. If this occurred, the drag associated with dynein microtubule binding could decrease kinesin-based transport. These highly speculative possibilities highlight the importance of understanding the molecular mechanism of action of ciliobrevin.

The studies by Roossien et al. (2014) and Sainath and Gallo (2014) both noted that treatment with ciliobrevin D alone induces some degree of retraction of the distal axon in established axons that were actively extending. Ciliobrevin D was also found to decrease the microtubule content of distal axons (Sainath and Gallo, 2014) and mitochondria exhibited net retrograde displacements in the distal-most segment of the axon (Roossien et al., 2014). When myosin II activity was inhibited using blebbistatin (Roossien et al., 2014), or actin filaments depolymerized using latrunculin-A (Sainath and Gallo, 2014), growth cones advanced faster and underwent collapse and exhibited retrograde cytoplasmic evacuation, respectively. Interestingly, treatment with ciliobrevin D partially countered both of these effects. These observations are generally consistent with the notion of antagonistic relationships between the actomyosin system and dynein (Ahmad et al., 2000; Myers et al., 2006). Further suggestions that dynein may be involved in the regulation of the axonal actin cytoskeleton arise from the investigation of the reorganization of actin filaments in axons undergoing Wallerian degeneration following severing from the cell body (Sainath and Gallo, 2014). During degeneration the actin filament content of axons was found to increase, and treatment with ciliobrevin D prevented the increase in axonal actin filaments while promoting the morphological degeneration of axons. These observations suggest an antagonistic role for dynein in axon degeneration, possibly through the regulation of the actin cytoskeleton which may have a protective modulatory role in the timing of the ensuing degeneration.

Firestone et al. (2012) found that ciliobrevin treatment resulted in the loss of pre-existing microtubules in mitotic spindles, but did not affect microtubules in non-mitotic NIH-3T3 cells. As noted above, Sainath and Gallo (2014) observed that ciliobrevin D treatment resulted in decreased levels of microtubules in the distal axon, and also altered the relative levels of tubulin post-translational modifications. While at first glance it may be surprising that the microtubules of a post-mitotic neuron would behave similarly to those of a mitotic cell in response to ciliobrevin treatment, this observation is consistent with the notion that neurons utilize aspects of the mitotic microtubule apparatus to extend their axons (Ferhat et al., 1998a,b; Buster et al., 2003; Nadar et al., 2008; Falnikar et al., 2011; Lin et al., 2012). However, Firestone et al. (2012) did not observe abnormal subcellular targeting of the p150^Glued^ dynactin subunit following treatment with ciliobrevins. In contrast, ciliobrevin D treatment resulted in the redistribution of p150^Glued^ to linear structures in axons (Sainath and Gallo, 2014), presumably mitochondria, indicating differences in the effects of ciliobrevin treatment on the distribution of p150^Glued^ in sensory neurons relative to NIH-3T3 cells.

## Concluding remarks

**Figure 3:**
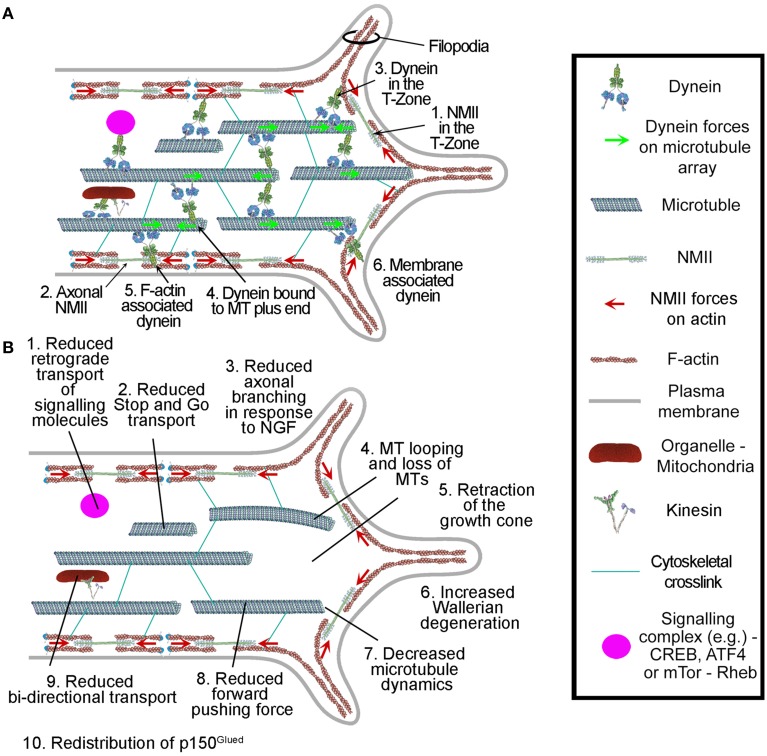
**Summary of the roles of dynein in axons and growth cones**. **(A)** Dynein localization and interactions in axons and growth cones. **(B)** Reported effects of dynein depletion/inhibition on axons and growth cones. All but items 2 and 7 were observed through chronic manipulation of dynein and also using ciliobrevins. The effects of ciliobrevins on stop-and-go transport and microtubule plus tip dynamics, items 2 and 7, remain to be directly determined. The role for dynein in axon degeneration, item 6, is based solely on the use of ciliobrevin D and requires confirmation though additional approaches.

The multifaceted functionality of dynein in neurons (Figure 3) makes it difficult to interpret the results of experiments involving the manipulation of dynein levels and function. Further complicating this problem has been the lack of reagents that are capable of acutely and locally disrupting dynein. For example, knocking down or overexpressing dynein affects the extension of axons, but because these approaches are chronic and global it has been difficult to resolve if dynein is playing a direct role in elongation at the growth cone, along the axon shaft or as the result of disrupting the trafficking of materials to and from the cell body. The strength of ciliobrevins is that they allow for acute and localized inhibition of dynein function. Nonetheless, while ciliobrevins are an attractive tool, cautionary notes ought to be taken into consideration. Dynein belongs to the AAA+ ATPase family of proteins, which mediate a variety of cellular processes. Firestone et al. (2012) did not detect effects on ciliobrevins on two other AAA+ ATPase proteins (p97 and Mcm2-7). However, given the diversity of this molecular family, further scrutiny will be required to rule out potential off target effects. Similarly, continued screening for possible effects of ciliobrevins on additional motor proteins is warranted. The bidirectional effects of ciliobrevins on the axonal transport of organelles may be due to the previously noted inter-dependence of antero and retrograde mechanisms, but may also reflect off target effects. The importance of this particular question in the field underscores the need for a tool that more confidently separates the action of individual motor subtypes in an acute and local fashion. Whether the ciliobrevins accomplish this degree of specificity is still unclear and will remain a limitation of their use until further insight into their action is gained. The bidirectional effects of inhibiting dynein function on transport mechanisms thus ought to be considered in the interpretation of experimental results which may be an indirect consequence of altered anterograde transport and not directly attributable to dynein *per se*. Alternatively, dynein can also serve to organize aspects of signal transduction mechanisms (Clippinger and Alwine, 2012; Kim et al., 2014; Wang et al., 2015). Thus, the effects of inhibiting dynein, even if acutely, may ultimately be due to its contribution to signaling mechanisms independent of its direct roles in the regulation of microtubules, an interpretational aspect that ought to be considered. For example, the inhibition of axon branching in response to NGF by ciliobrevin D (Sainath and Gallo, 2014) may in part be due to a role for dynein in organizing mTOR-Rheb signaling (Clippinger and Alwine, 2012), which is required for NGF-induced branching (Spillane et al., 2012). Continued analysis of the cellular effects of ciliobrevins will provide further insights into the degree of specificity of action of these compounds, and provide a richer landscape for the interpretation of results addressing the cellular functions of dynein. Specific important questions to answer going forward are as follows: Does ciliobrevin block the ability of ATP to bind dynein and thus leaves dynein in a nucleotide free conformation where it is bound tightly to the microtubule? Does it bind to dynein and locks it into an inactive conformation that mimics the ADP bound state, which again would cause it to associate tightly with microtubules? Does it bind to dynein and induces it to assume a transition state that mimics the active conformation, where dynein is weakly attached to the microtubule? Or does it does it bind to dynein and cause it to disassociate from the cargo? While some of these possibilities seem more plausible than others, clear answers to these questions will help in the interpretation of studies that use ciliobrevin, though it is likely that caution will continue to be needed in the interpretation of future studies. In sum, ciliobrevins are an important new tool as they now allow tight spatial and temporal manipulation of dynein. Nonetheless, ciliobrevins come with their own interpretational issues; they still remain to be fully characterized and off target effects remain a concern.

## Author contributions

DR, KM, and GG all continued equally to the writing of this manuscript. KM and GG share senior authorship.

### Conflict of interest statement

The authors declare that the research was conducted in the absence of any commercial or financial relationships that could be construed as a potential conflict of interest.
